# Urinary metabolomic profiling of a cohort of Colombian patients with systemic lupus erythematosus

**DOI:** 10.1038/s41598-024-60217-0

**Published:** 2024-04-25

**Authors:** Alejandra Rojo-Sánchez, Ada Carmona-Martes, Yirys Díaz-Olmos, Mary Santamaría-Torres, Mónica P. Cala, Erick Orozco-Acosta, Gustavo Aroca-Martínez, Leonardo Pacheco-Londoño, Elkin Navarro-Quiroz, Lisandro A. Pacheco-Lugo

**Affiliations:** 1 Life Sciences Research Center, School of Basic and Biomedical Sciences, Universidad Simón Bolívar, Barranquilla, Colombia; 2 Metabolomics Core Facility-MetCore, Vice-Presidency for Research, Universidad de los Andes, Bogotá, Colombia; 3 Health Sciences Division, Medicine Program, Universidad del Norte, Barranquilla, Colombia; 4 Clínica de la Costa, Barranquilla, Colombia

**Keywords:** Kidney, Metabolomics, Bioinformatics, Mass spectrometry, Metabolomics, Proteomic analysis, Biochemistry, Computational biology and bioinformatics, Biomarkers

## Abstract

Systemic lupus erythematosus (SLE) is an autoimmune and multisystem disease with a high public health impact. Lupus nephritis (LN), commonly known as renal involvement in SLE, is associated with a poorer prognosis and increased rates of morbidity and mortality in patients with SLE. Identifying new urinary biomarkers that can be used for LN prognosis or diagnosis is essential and is part of current active research. In this study, we applied an untargeted metabolomics approach involving liquid and gas chromatography coupled with mass spectrometry to urine samples collected from 17 individuals with SLE and no kidney damage, 23 individuals with LN, and 10 clinically healthy controls (HCs) to identify differential metabolic profiles for SLE and LN. The data analysis revealed a differentially abundant metabolite expression profile for each study group, and those metabolites may act as potential differential biomarkers of SLE and LN. The differential metabolic pathways found between the LN and SLE patients with no kidney involvement included primary bile acid biosynthesis, branched-chain amino acid synthesis and degradation, pantothenate and coenzyme A biosynthesis, lysine degradation, and tryptophan metabolism. Receiver operating characteristic curve analysis revealed that monopalmitin, glycolic acid, and glutamic acid allowed for the differentiation of individuals with SLE and no kidney involvement and individuals with LN considering high confidence levels. While the results offer promise, it is important to recognize the significant influence of medications and other external factors on metabolomics studies. This impact has the potential to obscure differences in metabolic profiles, presenting a considerable challenge in the identification of disease biomarkers. Therefore, experimental validation should be conducted with a larger sample size to explore the diagnostic potential of the metabolites found as well as to examine how treatment and disease activity influence the identified chemical compounds. This will be crucial for refining the accuracy and effectiveness of using urine metabolomics for diagnosing and monitoring lupus and lupus nephritis.

## Introduction

Systemic lupus erythematosus (SLE) is a chronic autoimmune disease characterized by global loss of self-tolerance and subsequent multiple-organ inflammation, which results in several relapsing–remitting clinical manifestations^1^. One of the main complications associated with SLE is lupus nephritis (LN), which occurs in approximately 50% of patients with SLE and is one of the main causes of morbidity and mortality secondary to SLE^2^. Therefore, early diagnosis of renal disease in patients with SLE is essential. Currently, renal biopsy is the gold standard for LN diagnosis and classification^3^. However, this invasive method is impractical for monitoring real-time kidney involvement and cannot predict whether patients will respond to treatment^4^. Therefore, it is necessary to study and identify new noninvasive biomarkers that can be used to guide renal biopsy, enable accurate prediction of relapse periods, monitor treatment response, and identify disease activity levels.

Metabolomics is an consolidated field defined as “the measurement of all low-weight molecules in a biological specimen to create diagnostic profiles with a higher number of metabolites than the profiles developed by standard clinical laboratory techniques.” Thus, metabolomics covers all the biological processes and metabolic pathways involved^5^. In recent years, metabolomics has been extremely useful for differentiating between sick and healthy individuals using noninvasive biological fluids, and multiple potential new diagnostic biomarkers of diseases have been identified^6^.

Metabolomics holds promise as a diagnostic and screening tool, enabling the identification of potential biomarkers across diverse pathological contexts, as well as offering novel insights into the underlying disease mechanisms^7^. Some reports describe metabolomics as an important tool for discriminating SLE patients with kidney involvement from SLE patients without kidney involvement^8^. However, few studies have explored the metabolomic profiles of SLE and LN in urine, a biological fluid considered a liquid biopsy of the kidney that can accurately reflect kidney imbalances in SLE patients.

In this study, we explored the usefulness of metabolomics as a way of identifying metabolites in the urine of a cohort of Colombian individuals with SLE and LN. Our findings revealed evident differences in the metabolic profiles of the study groups, with apparent changes in the metabolism of metabolic pathways such as those of fatty acids, bile acids, and amino acids.

## Materials and methods

### Study design and participants

This study was authorized by the Research Ethics Committee of Universidad Simón Bolívar (The project was formalized through Project Approval Record No. 00220 on May 24, 2019). All patients gave informed consent before sample collection and all methods were performed in accordance with the current guidelines and regulations for studies involving humans. A total of 50 individuals were included: 17 patients diagnosed with SLE and no kidney involvement (SLE group), 23 patients diagnosed with focal and diffuse LN (LN III/IV group: 6 were classified as LN stage III, while the remaining 17 were classified as LN stage IV.), and 10 clinically healthy individuals (HC, group) with no history of autoimmune disease or kidney involvement.

Patients were classified as having SLE based on the American College of Rheumatology (ACR) criteria^9^. Patients with clinical and laboratory alterations fulfilling the screening guidelines for LN by the ACR—LN defined as continuous proteinuria > 0.5 g/24 h, urinary sediment alteration, and/or progressive kidney dysfunction—were classified within the LN group, and their histopathological diagnosis was confirmed by renal biopsy. Patients diagnosed with other autoimmune or immunological diseases were excluded.

### Sample collection and preparation

Urine samples were collected from each participant in a sterile polypropylene container during the first hours of the morning. After collection, the samples were centrifuged at 1500×*g* for 15 min at 4 °C to remove the urine sediment. To prevent bacterial contamination and interference with downstream metabolomics analyses, sodium azide was added at a final concentration of 10 mM, and the samples were divided into single-use 1000-μL aliquots. Following sample collection, all procedures were conducted on ice, and the aliquots were stored at − 80 °C until chromatography was performed.

Quality control (QC) samples were prepared by mixing equal volumes of each urine sample. The QC samples were treated in the same manner as the analytical samples and were introduced at the beginning of the experiment to balance the systems, then they were injected every 10 samples in the analytical sequence to evaluate the reliability of the large-scale metabolomics analysis.

### Metabolomic analysis by GC–QTOF–MS

The urine samples were vortexed for 1 min for homogenization. Subsequently, 50 units of urease solution were added to 100 μL of urine, and the samples were incubated at 37 °C for 1 h. After incubation, 400 μL of cold methanol (− 20 °C) was added to each urine sample, and the samples were vortexed again for 5 min and immediately incubated at − 20 °C for 20 min. Subsequently, the samples were centrifuged at 31,208×*g* at 4 °C for 15 min.

In the next step, 100 μL of the previous preparations was dried using a SpeedVac (Thermo Scientific). Furthermore, 30 μL of O-methoxyamine in pyridine (15 mg/mL) was added, and the preparation was vortexed at 986×*g* for 5 min, followed by incubation in the dark at room temperature for 16 h. Silylation was conducted by adding 30 μL of *N,O*-bis(trimethylsilyl)fluoroacetamide to 1% trimethylchlorosilane, followed by vortex agitation for 5 min and incubation at 70 °C for 1 h. After the preparation, 80 μL of methyl stearate in heptane was added as an internal standard (10 mg/L).

The data were collected using gas chromatography (Agilent Technologies, Palo Alto, CA, USA) coupled with a time-of-flight mass spectrometer (TOF–MS; Agilent Technologies 7250 GC/Q-TOF, Agilent Technologies, Palo Alto, CA, USA). Furthermore, 1 µL of the sample derivatives was injected into an HP-5MS column (30 m × 0.25 mm × 0.25 µm) (Agilent Technologies, USA) considering a split ratio of 30 and a continuous flow of 0.7 mL/min. The oven temperature varied from 60 (1 min) to 325 °C (10 min) at 10 °C/min. Mass spectra were registered at 70 eV in full-scan mode, with m/z values within the 50–600 range. The temperature of the transfer line connected to the detector, that of the source filament, and that of the quadrupole were maintained at 280 °C, 230 °C, and 150 °C, respectively.

### Metabolomic analysis by LC–QTOF–MS

For this step, 200 µL of water was added to 100 µL of each sample, followed by vortex agitation at 986×*g* for 1 min. Finally, the samples were centrifuged at 27,800×*g* at 4 °C for 10 min. The samples were analyzed using a liquid chromatography (LC) system (Agilent Infinity 1260) coupled to a quadrupole TOF‒MS (QTOF‒MS) analyzer (Agilent 6545) with electrospray ionization (ESI; Agilent Jet Stream ESI source). To achieve this goal, 2 μL of sample was injected into a column (50 × 2.1 mm, 1.8 µm; ZORBAX Eclipse Plus C18; Agilent, USA) at 40 °C. Gradient elution consisted of 0.1% (v/v) formic acid in water (Mobile Phase A) and 0.1% (v/v) formic acid in acetonitrile (Mobile Phase B) with a continuous flow of 0.5 mL/min. MS detection was conducted with positive and negative ion ESI and 100 to 1100 m/z full-scan mode^10^. The QTOF instrument was operated in 4 GHz (high-resolution) mode. Two reference masses were used throughout the analysis for mass correction: m/z 121.0509 (C_5_H_4_N_4_ + H), m/z 922.0098 (C_18_H_18_O_6_N_3_P_3_F_24_ + H)^+^ in positive ion mode and m/z 112.9856 (C_2_O_2_F_3_ + NH_4_)^−^, m/z 1033.9881 (C_18_H_18_O_6_N_3_P_3_F_24_ + FA − H)^−^ in negative ion mode. MS/MS data acquisition was conducted in data-dependent acquisition mode using a quality control sample at different collision energies: 20 and 40 eV.

### Data treatment

To prepare the data for statistical analysis, we applied Pareto scaling and logarithmic transformation. The raw data obtained by LC–QTOF–MS were deconvoluted, aligned, and integrated using Agilent MassHunter Profinder B.10.0. software. This was followed by manual inspection to remove background noise and signals from the reagent blank and the column. For GC-QTOF-MS, the data were deconvoluted, aligned, and integrated using Agilent Unknowns Analysis B.10.0 software, MassProfiler Professional v15 software, and Agilent MassHunter Quantitative Analysis B.10.00 software, respectively.

For both LC and GC, the raw data obtained was normalized by the total area to reduce natural variability in metabolite concentrations in urine samples, facilitating a more accurate comparison of individual metabolite concentrations. Additionally, for GC data, normalizations based on the internal standard were performed to correct for inherent instrument variation throughout chromatographic analysis. Subsequently, we applied a filter based on characteristic presence and reproducibility, selecting only the characteristics present in 80% of the samples with a quality control coefficient of variation (CV, %) < 20% (or 30% for GC data).

### Identification of metabolites

Metabolites obtained by LC were registered based on their monoisotopic mass, isotopic distribution, adduct formation, and molecular formula. To this end, online databases such as the Human Metabolome Database (http://hmdb.ca), KEGG (http://genome.jp/keg), MassBank (https://massbank.eu/MassBank/), Lipid MAPS (http://lipidmaps.org), and METLIN (http://metlin.scripps.edu) were searched using the CEU Mass Mediator tool (http://ceumass.eps.uspceu.es/). Subsequently, the MS/MS spectra obtained were compared with MS-DIAL 4.8 software to determine the identities of the metabolites (http://prime.psc.riken.jp/compms/msdial/main.html). For the GC data, the Fiehn GC–MS Metabolomics RTL Library was used, which considers coincidence in retention times, mass spectra, and retention indices considering fatty acid methyl ester (FAMES) to simplify metabolite identification^11^. For LC and GC, metabolites were reported with a confidence level based on the Metabolomics Standards Initiative (MSI) guidelines^12^, which determine five levels: Level 0, unambiguous 3D structure requiring isolation of pure compound; Level 1, matched with reference standard; Level 2, probable structure, corroborated by literature or database evidence; Level 3, putatively characterized compounds with matching molecular formula; and Level 5, metabolites only matching MS1 in online databases.

### Statistical analysis

We measured the quality of the system performance during analysis using SIMCA-P + 16.0 (Umetrics) software through principal component analysis (PCA). This multivariate approach enables the visualization of instrumental stability over time. Orthogonal partial least squares discriminant analysis (OPLS-DA) was subsequently conducted to model intergroup differences^13^.

Metabolites with statistically significant differential expression between the SLE and LN groups were identified using univariate statistical analysis (UVA), nonparametric *t* tests (Mann–Whitney *U* test) and Benjamini–Hochberg false discovery rate corrections were applied using MATLAB. In addition, data normality was assessed using the Shapiro–Wilk test and the Lilliefors test based on the Kolmogorov–Smirnov test, and the relationship between variances was determined with Levene’s test. The significant metabolites selected had to fulfill at least one of the following criteria: UVA: p < 0.05 (In the event that Benjamini–Hochberg false discovery rate corrections are applied; it will be specified in Supplementary Table S1 with an asterisk (*)) or MVA: variable importance for projection (VIP) > 1.

Fisher’s least significant difference (LSD) test was used to compare the concentration means of each significant metabolite between both groups. Finally, the predictive power of the differentially abundant metabolites was assessed using receiver operating characteristic (ROC) analysis and estimating the area under the curve (AUC) with MetaboAnalyst 5.0.

**Figure 1:**
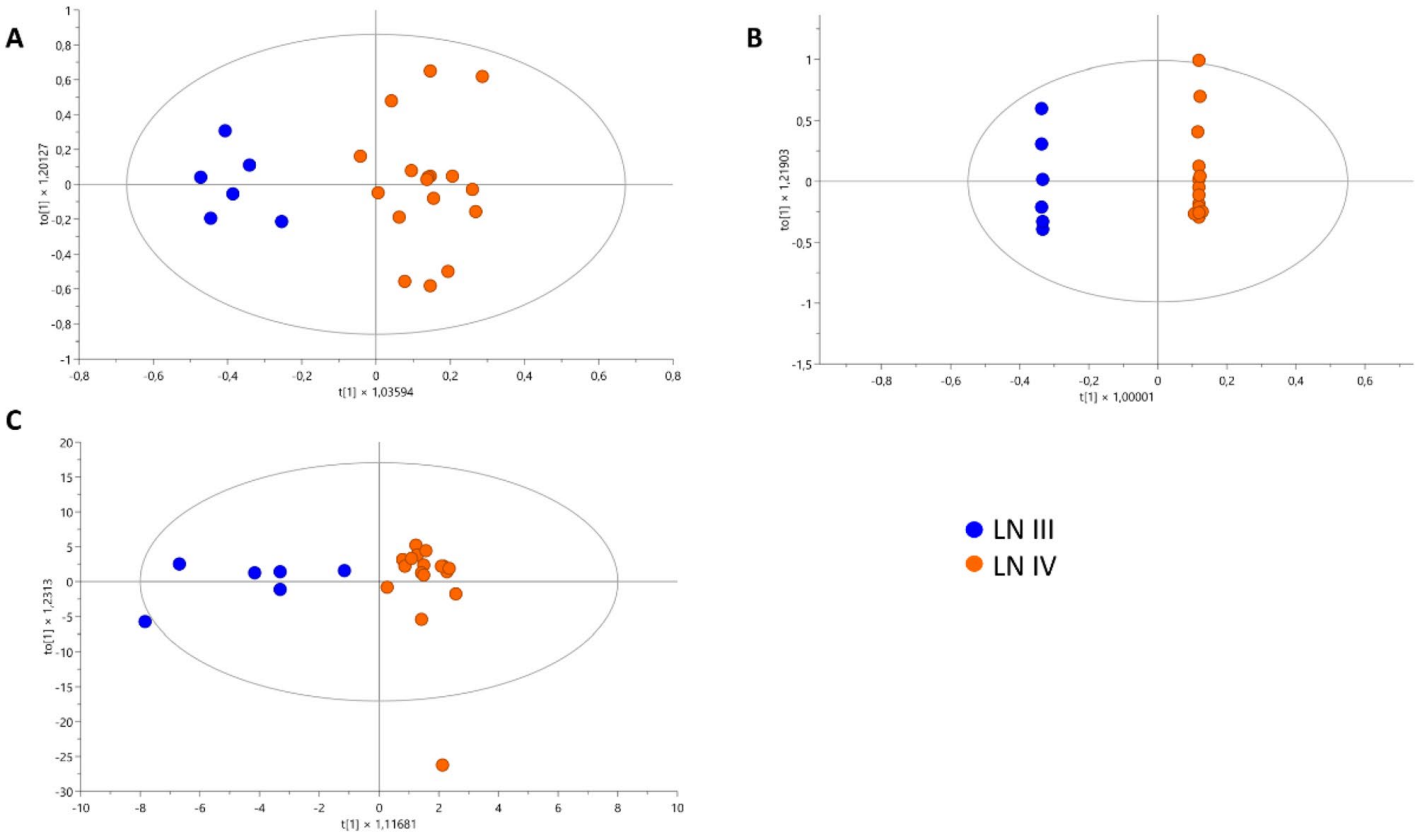
OPLS-DA Models with logarithmic transformation and Pareto scaling for the metabolomic profile in urine samples from LN III (blue points) and LN IV (orange points). (**A**) Untargeted metabolomics through LC–QTOF–MS (+): R^2^: 0.208 R^2^Y: 0.881 Q^2^: 0.288 cv-ANOVA = 0.169. (**B**) Untargeted metabolomics through LC–QTOF–MS (−): R^2^: 0.376 R^2^Y: 0.943 Q^2^: 0.74. cv-ANOVA = 3.85e−08. (**C**) Untargeted metabolomics through GC–QTOF–/MS: R^2^: 0.346 Q^2^: 0.197 cv-ANOVA = 0.386.

The study refrained from conducting a statistical comparison between LN III (n = 6) and LN IV (n = 17) groups due to concerns arising from the substantial imbalance in sample sizes (Fig. 1). This disparity raised questions about the statistical robustness and reliability of potential findings. With the LN III group significantly smaller than the LN IV group, engaging in statistical analysis would compromise statistical power, impairing the capacity to detect genuine differences and elevating the risk of false-negative results. Moreover, the limited LN III sample size fails to adequately represent the inherent diversity within each Lupus Nephritis class. Consequently, there is a challenge in capturing the true breadth of metabolic profiles within each group, introducing the potential for biased or skewed results. Additionally, the smaller LN III group increases the risk of overfitting statistical models to idiosyncrasies in the data, diminishing the overall robustness of any identified differences. Lastly, the findings may encounter difficulties in terms of external validation, which constitutes a future goal for this investigation.

### Ethics approval and consent to participate

This work was approved by the ethics committee of Simón Bolívar University (The project was formalized through Project Approval Record No. 00220 on May 24, 2019), and all subjects signed an informed consent prior to sample collection.

## Results

### Sociodemographic characteristics of the study population

**Figure 2:**
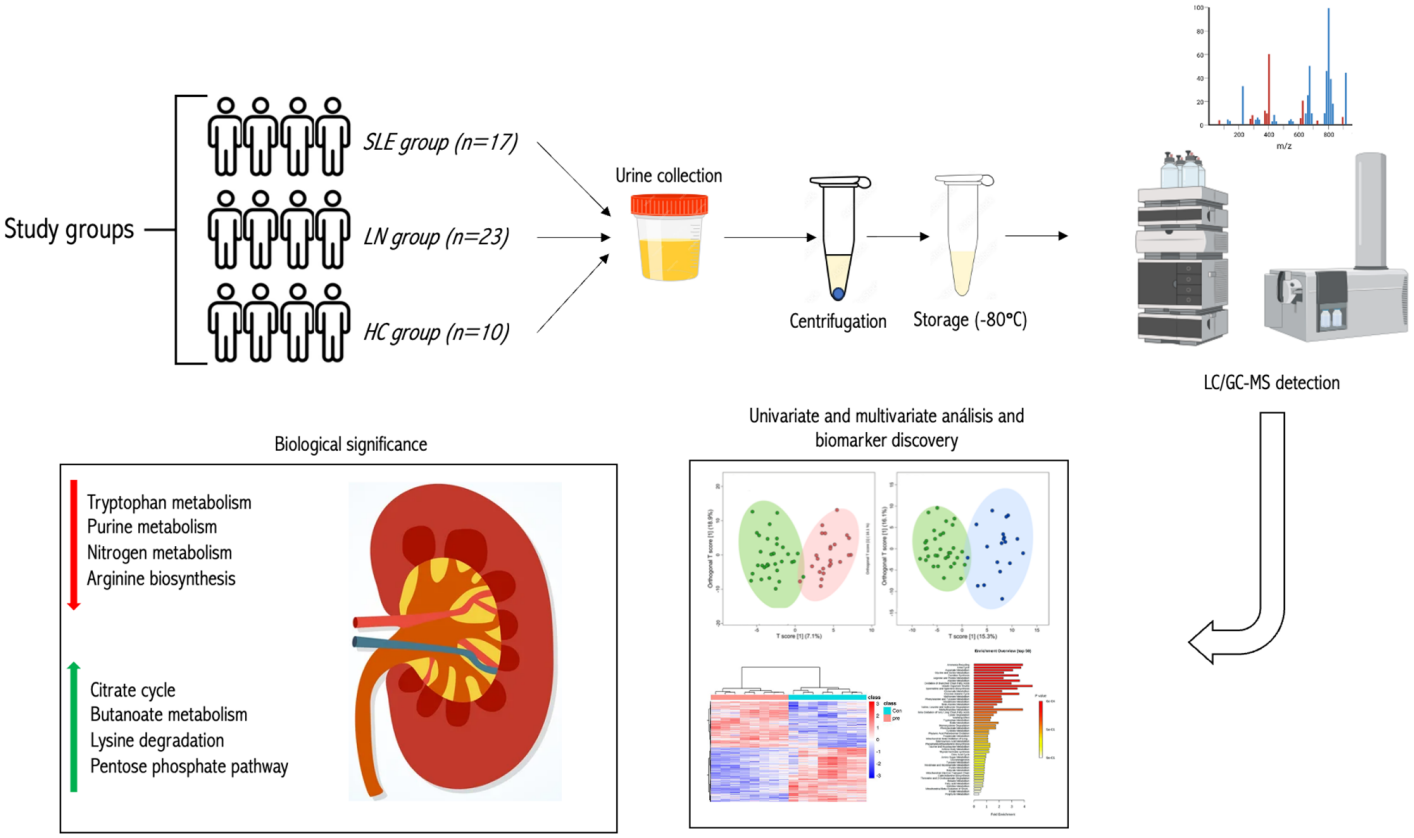
The workflow of urine biomarker discovery in SLE and lupus nephritis.

We used untargeted metabolomics to identify urine metabolites in 17 individuals with SLE, 23 individuals with LN, and 10 clinically healthy controls paired by age and sex, using liquid and gas chromatography coupled with mass spectrometry following a workflow for urine samples (Fig. 2).

Table 1 summarizes the baseline clinical characteristics of the study groups. In addition, clinical data such as antinuclear antibody presence, arthralgia, alopecia, protein level of the complement system, creatinine, proteinuria, blood urea nitrogen, urea, and creatinuria were included. Of the 50 study participants, 92% were female. This gender represented 94% of the SLE group, 87% of the LN group, and 100% of the healthy controls. Patients with SLE and LN were generally positive for antinuclear antibodies and hypocomplementemia, and most of them had a history of arthralgia and alopecia. The average creatinine levels in patients with SLE were significantly greater than those in patients with LN.

**Table 1:** Demographic characteristics of patients with SLE and LN at the time of urine sample collection.

Characteristics	SLE group	NL group	p-value
Sample number	17	23	–
Age	26.88 ± 4.96	32.13 ± 8.40	–
Sex (F:M)	16:1	19:4	–
Arthralgia (yes:no)	17:0	22:1	–
Alopecia (yes:no)	14:3	16:7	–
AntiDNA positive	17	23	–
ANAS positive	17	22	–
C4 (mg/dl)	7.99 ± 1.24	11.88 ± 13.74	0.188
C3 (mg/dl)	65.00 ± 15.76	58.65 ± 13.88	0.859
Mouth ulcers (yes:no)	0:17	8:15	–
SLICC	3.18 ± 1.24	2.74 ± 1.21	0.285
Creatinine (mg/dl)	1.96 ± 0.74	1.10 ± 0.62	0.001
Proteinuria (mg/dl)	0.77 ± 0.22	222.33 ± 64.58	0.000
BUN (mg/dl)	–	33.10 ± 20.46	–
Urea (mg/dl)	–	79.82 ± 40.23	–

### Multivariate statistical analysis of metabolites

**Figure 3:**
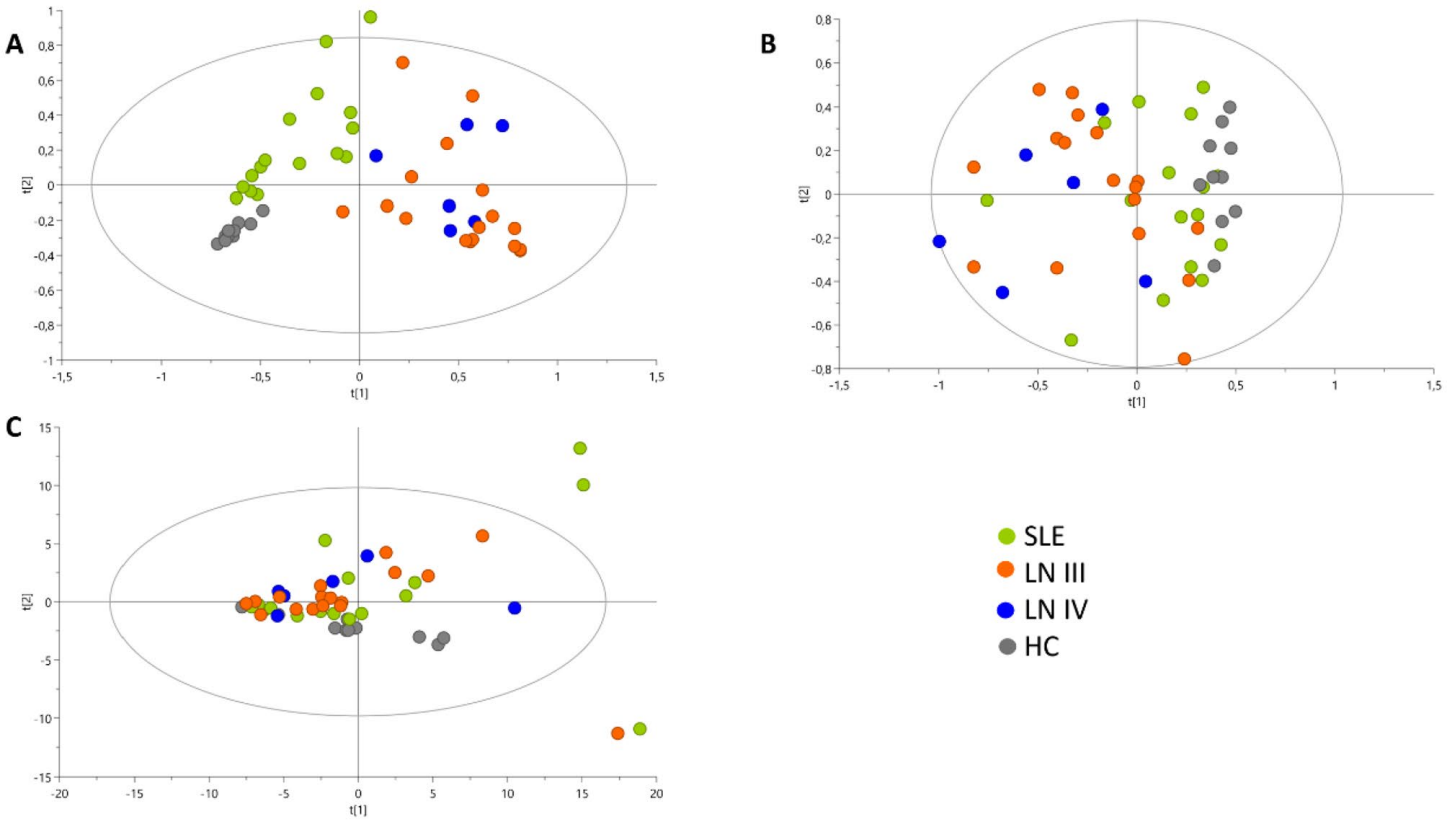
PCA scatter plots for the dataset filtered by presence and reproducibility. (Green points: SLE group; Blue points: LN III; Orange points: LN IV; Gray points: HC). (**A**) Untargeted metabolomics through LC–QTOF–MS (+): R^2^ = 0.682. Q^2^ = 0.341 with the first seven components. (**B**) Untargeted metabolomics through LC–QTOF–MS (−): R^2^ = 0.687. Q^2^ = 0.309 with first three components. and (**C**) untargeted metabolomics through GC–MS: R^2^ = 0.721. Q^2^ = 0.05 with first four components.

A data quality analysis was conducted before the statistical analysis of the differentially expressed metabolites. The quality control samples were pooled in PCA models to confirm the results and assess the performance of the analytical platforms used (Supplementary Fig. S1A–C), which suggested good stability and solid analytical performance of the platforms throughout the analysis. The control, SLE, and LN groups are illustrated in a PCA plot, which shows spontaneous biological separation between participants with no kidney involvement (controls and SLE) and patients with LN (LN III and LN IV). However, despite the trend toward separation, the data variability and intergroup overlap may suggest the influence of other biological factors (Fig. 3).

**Figure 4:**
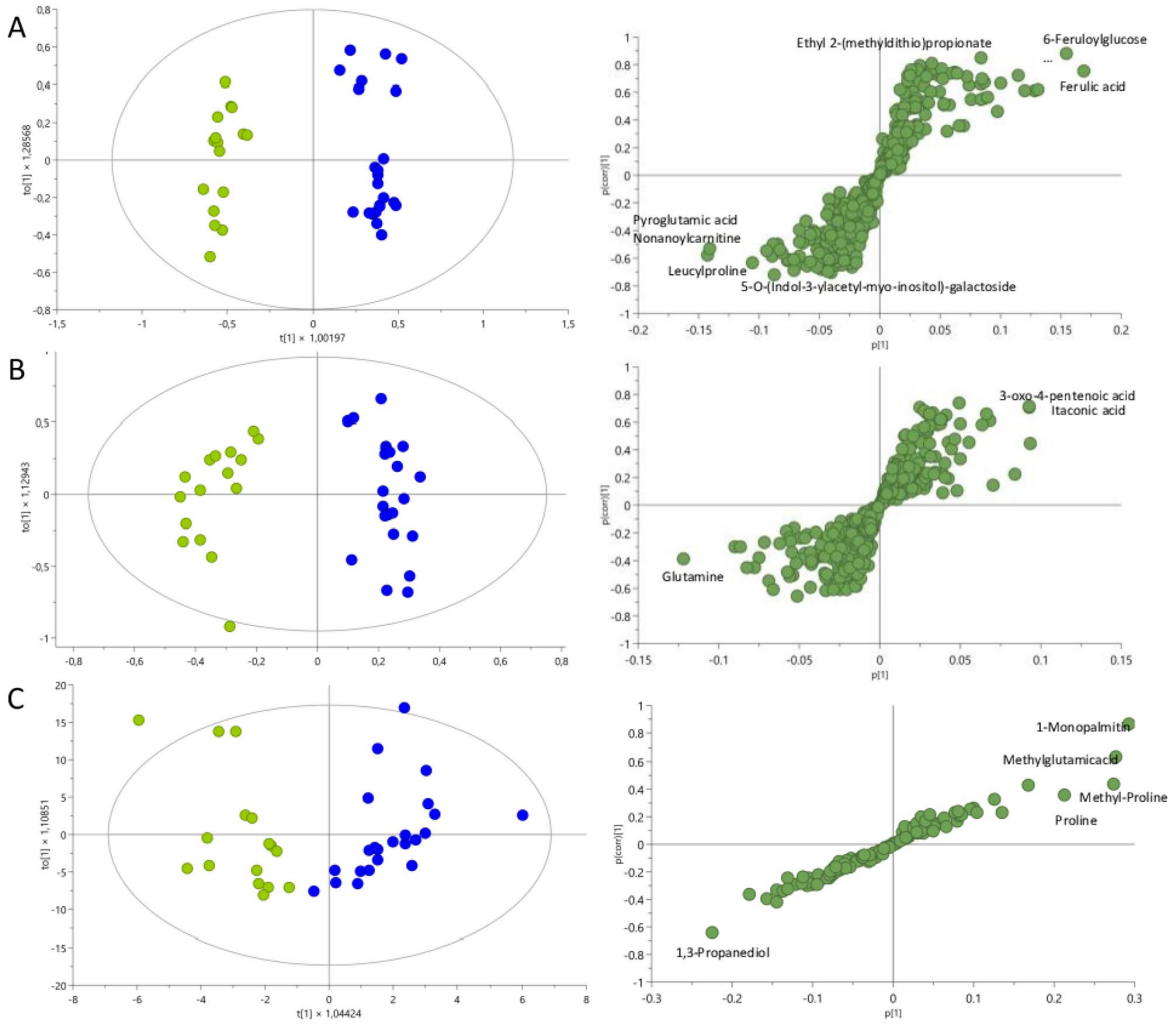
OPLS-DA models with logarithmic transformation and pareto scaling for the metabolomic profile in urine samples from SLE (green points) and LN (blue points) along with a loadings S-plot illustrating the metabolites significantly contributing to the variation between the compared groups. (**A**) Untargeted metabolomics through LC–QTOF–MS (+): R^2^: 0.380 R^2^Y: 0.969 Q^2^: 0.853 cv-ANOVA = 5.39e−12. (**B**) Untargeted metabolomics through LC–QTOF–MS (−): R^2^: 0.376 R^2^Y: 0.943 Q^2^: 0.74. cv-ANOVA = 3.85e−08. (**C**) Untargeted metabolomics through GC–QTOF–MS: R^2^: 0.470 R^2^Y: 0.769 Q^2^: 0.358 cv-ANOVA = 0.0201.

To improve the effects of sample classification, OPLS models were constructed to explore the differences between the SLE group and LN group (LN class III and IV). Figure 4 shows the score plots for each of these comparisons by LC–QTOF–MS in positive and negative ion modes, as well as GC–QTOF–MS. The models reflect adequate distinction between groups; however, when comparing patients with SLE, and patients with LN class III and class IV (Fig. 4A,B), data separation is better achieved with LC–QTOF–MS than with GC–QTOF-– (Fig. 4C). Additionally, each figure is accompanied by its corresponding loadings S-plot, offering visual insight into the metabolites contributing to the observed separation. These plots reveal that the separation is driven by a limited number of metabolites. The discrepancy between techniques was to be expected as each one has its own set of advantages and limitations. The differences in the physical and chemical properties of these techniques, such as differences in mobile phases, stationary phases, temperatures, and detectors, among other factors, can lead to variations in results even when analyzing the same samples. Combining these methods in a complementary manner can enhance the overall analytical capabilities and provide more comprehensive information about a sample´s metabolome. The discussion of this article was based on the combined results acquired through the use of both techniques. A combined strategy consisting of MVA with a VIP threshold > 1 and UVA with a significance value (*p* value) < 0.05 was applied to identify specific distinctive metabolites. Overall, 50 metabolites that allowed for the distinction between the SLE and LN III/IV groups were identified. From a global perspective, LC–QTOF–MS identified a greater number of relevant metabolites, which belong to a greater number of compound groups, such as amino acids, bile acids, alcohols, carbohydrates, fatty acids, glycerophospholipids, hydroxy acids, imidazoles, indoles, organic acids, organic disulfides, phenylpropanoids, prostaglandins, purines, and pteridines. However, GC–QTOF–MS could only identify statistically significant metabolites from the organic acid, amino acid, nucleoside, carboxylic acid, glycerolipid, and carbohydrate families (Supplementary Table S1 and Table 2). Notably, the same metabolite was not significantly differentially expressed between the two methods.

**Table 2:** Comparison is between SLE and LN.

Metabolite	AUC	Se (%)	Sp (%)
Monopalmitin	1	100	100
Methyl-Glutamic Acid	1	100	100
Glycocholic Acid	0.947	90	90
5-O-(Indol-3-ylacetyl-myo-inositol)-galactoside	0.940	90	90
Indol-3-ylacetyl-myo-inositol-arabinoside	0.902	90	80
Pteridine	0.891	90	80
Methyladenine	0.883	80	80
Allantoin	0.880	80	100
Creatinine	0.853	80	80
Leucylproline	0.851	70	90
Nonanoylcarnitine	0.842	80	90
Benzoic acid	0.837	80	90
Pyroglutamic acid	0.829	80	80
Gluconic acid	0.826	80	70
Hydroxyanthranilic acid	0.819	80	80
Valine	0.812	90	70
Erucamide	0.800	70	90

List of metabolites with the best predictive power.

*AUC* area under the curve, *Se* sensitivity, *Sp* specificity.

### Metabolic alterations in patients with systemic lupus erythematosus and lupus nephritis

**Figure 5:**
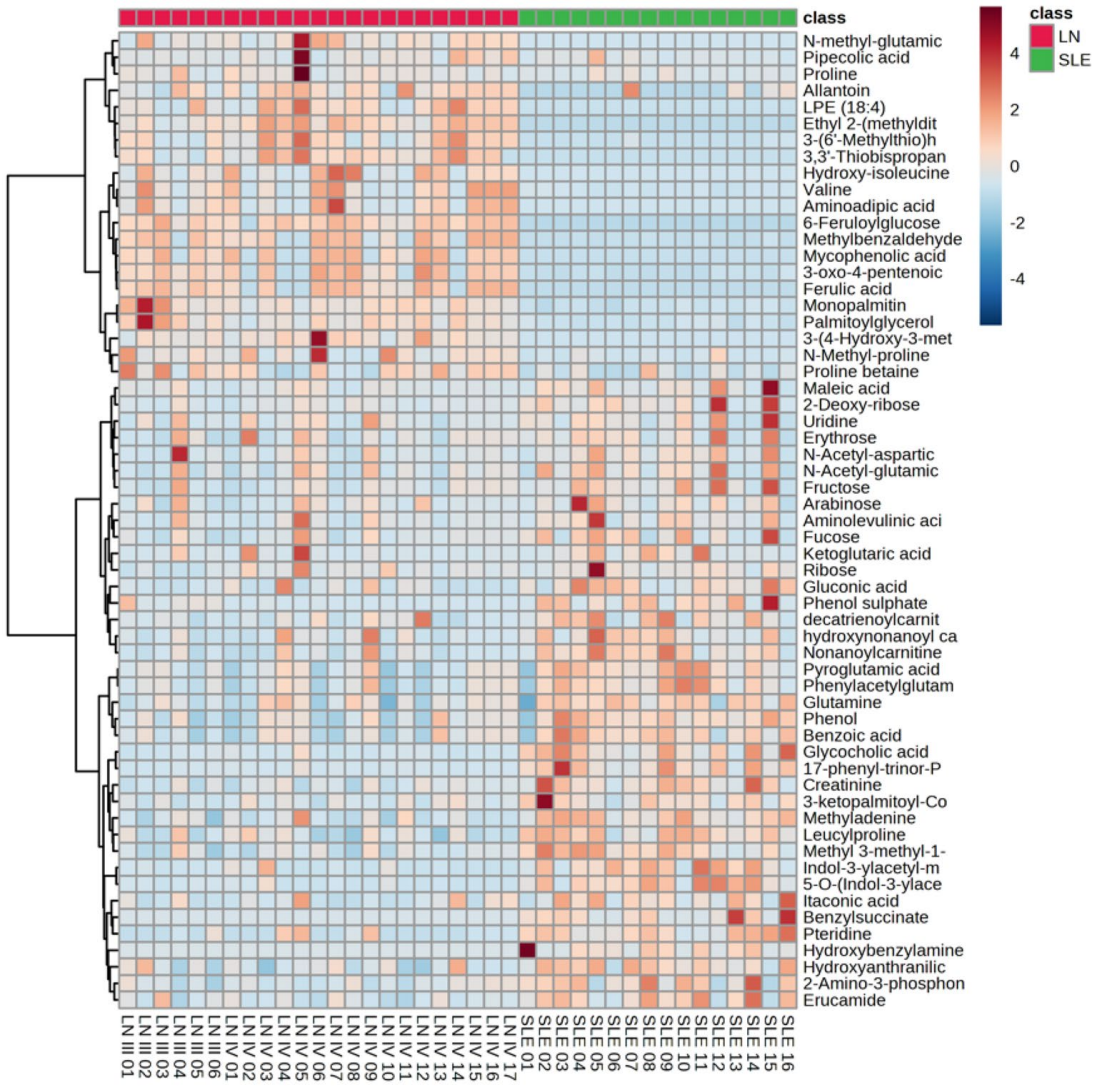
Heat map for the 59 metabolites identified by LC-QTOF-MS and GC/MS-QTOF using the KEGG database Comparison between the relevant metabolites identified in the LN versus SLE urine samples. On the top right is the colorimetric scale: if the color tends to dark red, then the metabolite's concentration was increased; if the color tends to dark blue, then the metabolite's concentration was decreased. The red and green boxes on the upper right correspond to the LN group (Lupus Nephritis; n = 17; red) and SLE group (systemic lupus erythematous; n = 16; green). On the left side of the heatmap is the clustering division. Results obtained by the cluster analysis performed with MetaboAnalyst 5.0.

**Figure 6:**
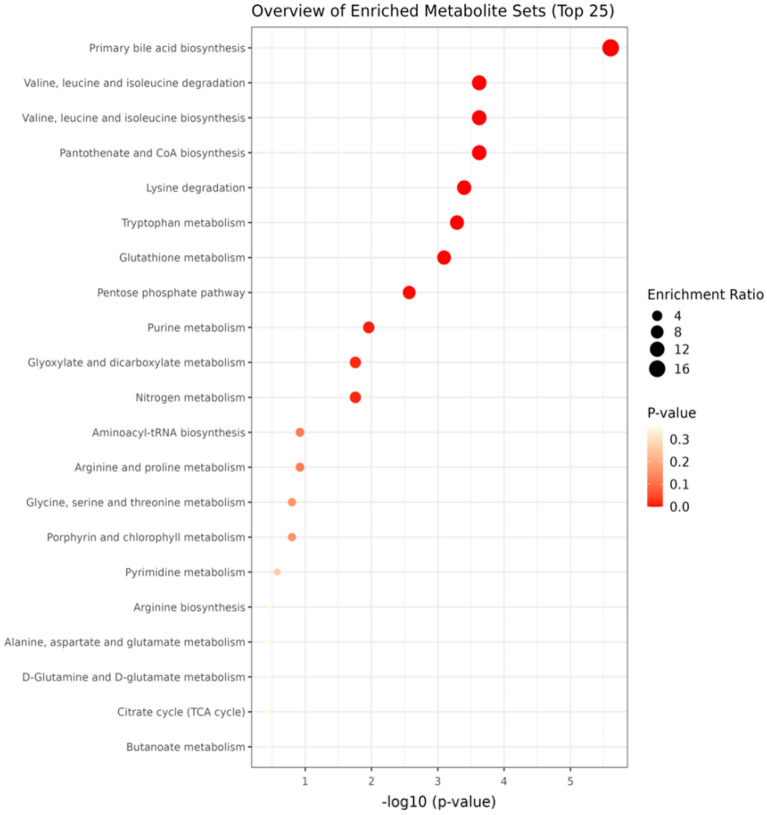
Metabolite set enrichment analysis using MetaboAnalyst 5.0. The size of the circles indicates the Enrichment Ratio, while the color represents the p-value, therefore, on the x-axis, higher value represents more significant associations between the metabolite set and the pathway.

Based on the above results, heatmaps were generated to observe metabolite behavior in the study groups (Fig. 5). Subsequently, Fisher’s LSD test was conducted, revealing significant differences between the two groups (SLE and LN) and leading to the identification of 42 metabolites with notable distinctions. Additionally, a metabolite set enrichment analysis (MSEA) was conducted to assess the relationship between the concentration of each metabolite and potential metabolic pathways. The metabolic pathways showing the highest differential expression between the LN group and the SLE group included primary bile acid biosynthesis, branched-chain amino acid (BCAA) synthesis and degradation, pantothenate and coenzyme A (CoA) biosynthesis, lysine degradation, and tryptophan metabolism (Fig. 6).

### Identification of potential biomarkers

**Figure 7:**
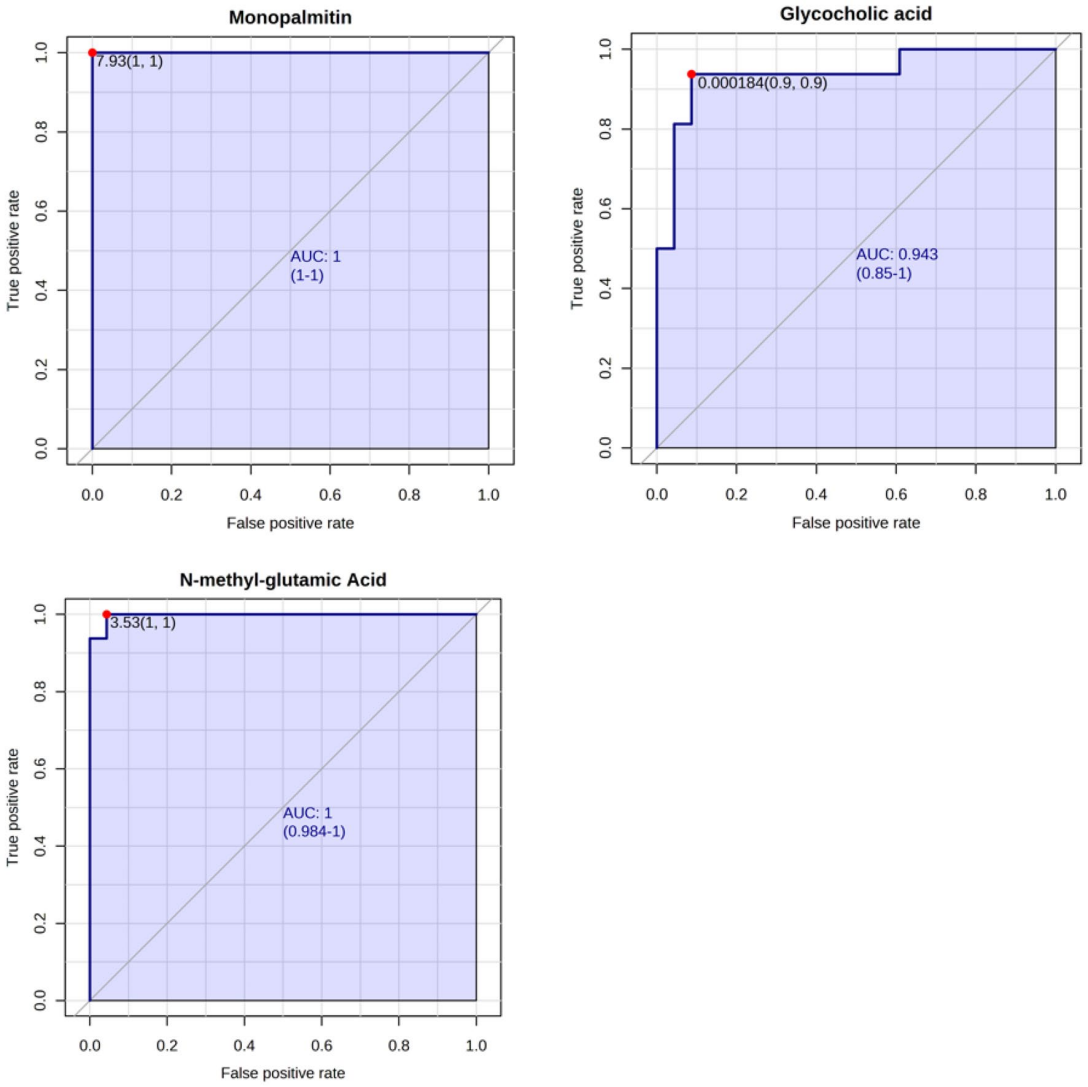
ROC curves of the three metabolites with the best predictive value. (**A**) Monopalmitin; (**B**) glycocholic acid; and (**C**) N-methyl-glutamic Acid. AUC: the sensitivity and specificity are represented on the y and x axes, respectively. The area under the curve (AUC) shows in blue, with 95% confidence intervals (CI).

In this study, the relevant metabolites were tentatively assigned based on level 2 confidence, utilizing a combination of molecular formula determination through high-resolution mass spectrometry and MS/MS fragmentation patterns compared against existing databases and in-silico prediction tools. Further studies, including comparison with authentic standards and are necessary to confirm the annotations. The usefulness of discrepant metabolites for LN prediction was assessed using receiver operating characteristic (ROC) analysis, and the performance of metabolites as possible LN biomarkers was estimated using area under the curve (AUC) values. The latter allowed for the identification of 18 metabolites with AUC values greater than 0.8 (Table 2). The metabolites with the highest ability to discriminate between patients with SLE and patients with and without kidney involvement were monopalmitin (AUC = 1, 95% IC 1–1), methyl-glutamic acid (AUC = 0.984, 95% IC 0.984–1), and glycolic acid (AUC = 0.943, 95% IC 0.85–1) (Fig. 7).

### Correlations between urinary metabolite levels and kidney function indicators

Finally, a Pearson’s correlation analysis was conducted to determine the relationship between the clinical and demographic characteristics of patients with SLE and LN and between the monopalmitin, methyl-glutamic acid, and glycolic acid values (Table 3), the metabolites with the highest predictive power. No significant associations were found between the concentrations of these metabolites and variables of interest, such as age, complement concentration, systemic lupus international collaborating clinics (SLICC) score, creatinine level, or proteinuria.

**Table 3:** Analysis of Pearson correlations between the metabolites of interest and the clinical and demographic variables.

	(1)	(2)	(3)	(4)	(5)	(6)	(7)	(8)
Age (1)								
C4 (2)	− 0.3436**							
C3 (3)	− 0.1032	0.1585						
SLICC_ACR (4)	0.2585	− 0.0316	− 0.1167					
Creatinine (5)	− 0.1003	− 0.213	− 0.1822	− 0.1664				
Proteinuria (6)	0.0008	0.0644	− 0.2599	− 0.0965	0.0021			
N_methyl_glutamic Acid (7)	0.2505	− 0.2598	0.2351	− 0.2199	− 0.1836	− 0.0365		
Monopalmitin (8)	0.1227	− 0.3606**	0.094	− 0.2465	− 0.0584	− 0.0205	0.3482**	
Glycocholic acid	− 0.2138	0.0424	0.0473	− 0.1102	0.3777**	− 0.201	− 0.2674	− 0.4746**

The range of these correlation coefficients is from − 1 to + 1, and they measure the strength of the linear relationship between the variables. Values in bold and underlined are indicative of statistical significance.

**0.05 Significant two sided.

## Discussion

Metabolomics has shown considerable potential for identifying new molecules associated with the occurrence of several pathological disorders and even for classifying patients based on the degree to which a specific organ is affected. By leveraging the advantages of this technique, we explored the usefulness of metabolomics for identifying the metabolic profiles associated with kidney damage in urine samples from patients with SLE using LC–QTOF–MS and GC–QTOF–MS to identify small molecules that may contribute to the noninvasive diagnosis of LN.

In the context of a metabolomics study utilizing urine samples for SLE and LN the issue of normalization becomes particularly complex, especially in the presence of kidney damage associated with LN. Normalizing metabolite levels is a critical step to ensure accurate comparisons and interpretations, but when kidney function is compromised, as in LN, traditional normalization methods may face challenges. Correlating metabolite levels to creatinine, a commonly used approach, may not be sufficient in diseases like LN, where creatinine itself could be influenced by renal dysfunction, so an additional normalization strategy, useful MS signal was applied^14^. In this study we found that even after normalization, there were many metabolites that increased or decreased between the SLE and LN groups, which suggests that the observed metabolomic changes may not solely result from kidney leakage. This could imply the presence of broader systemic metabolic alterations associated with the disease, extending beyond renal dysfunction.

LC–QTOF–MS and GC–QTOF–MS allowed for the identification of 50 differentially expressed metabolites, revealing a statistically significant difference between the SLE and LN groups. The urine samples from patients with LN yielded higher concentrations of certain metabolite groups, such as amino acids, lipids, and organic acids. We also identified an relevant statistical increase in the levels of monopalmitin and glutamic acid, as well as a decrease of glycolic acid—that could help discriminate between patients with SLE with and without kidney involvement; these metabolites are proposed as potential biomarkers for LN diagnosis.

Monopalmitin is a monoacylglycerol and is the final product of the intestinal digestion of dietary fats. Intestinal cells transform monoacylglycerols into triacylglycerols (TAGs), which are subsequently transported to the liver. Ouyang, et al. reported that lipid metabolites were considerably increased in the serum of patients with SLE compared with healthy individual^15^. In addition, Shin, et al. reported marked elevations in the concentrations of certain lipids, such as palmitoleic acid, oleic acid, and eicosanoic acid, in the plasma of patients with SLE compared with healthy individuals^16^. Some studies have also proposed the lipid nephrotoxicity hypothesis, in which TAG and fatty acid deposits in renal tissue induce glomerular diseases, such as LN^17^. In these cases, the inflammatory stress secondary to renal disease changes lipid homeostasis, thus increasing cholesterol absorption, decreasing cholesterol efflux, and changing cholesterol synthesis. Consequently, cholesterol accumulates in renal tissue and leads to renal failure^18^. Other studies have also reported an increase in sterol regulatory element-binding protein (SREBP) expression in patients with kidney damage, thus contributing to TAG and cholesterol accumulation and resulting in glomerulosclerosis and proteinuria^19^. Our study showed a significant increase in lysophosphatidylethanolamine glycerophospholipid (18:4) levels in patients with LN compared with patients with SLE (fold change: 297), as did 3-oxo-4-pentenoic fatty acid levels (fold change: 15.02), which supports the idea that dyslipidemia contributes to kidney damage in patients with SLE.

A hyperlipidemic environment is associated with lipid peroxidation. Oxidized low-density lipoprotein (LDL) directly damages podocytes through chemokine ligand 16 (CXCL16) by inducing the production of reactive oxygen species (ROS). Thus, high CXCL16 and oxidized LDL levels have been reported in the renal tissue of patients with different glomerular diseases^20,21^. Several studies have shown a close relationship between oxidative stress and inflammation and between oxidative stress and autoimmune responses in patients with SLE. These studies have emphasized that oxidized phospholipids and metabolites resulting from increased oxidative stress may act as antigenic epitopes in patients with SLE, thus enhancing excessive antibody production and significantly accelerating LN progression^22,23^.

For glutamic acid, glutaminase has been reported to promote Th17 cell proliferation and activation. The expression of this enzyme is regulated by the transcription factor cAMP-response element modulator (CREM), which is overexpressed in the T cells of patients with LSE and MRL/lpr mice prone to SLE^24^. Additionally, inhibition of this enzyme improves SLE activity in LMR/lpr mice^25,26^. Glutamate oxaloacetate transaminase 1 (GOT1) also enhances Th17 cell differentiation, and its selective inhibition also significantly decreases Th17 differentiation in murine T cells^27,28^. In our study, we found higher glutamic acid levels in participants with LN than in participants with SLE with no kidney involvement (fold change = 4292.06). These findings are consistent with previous studies that related glutamine metabolism to kidney damage. However, in addition to its role in facilitating the inflammatory process, increased glutamate levels may be explained by the fact that patients with terminal renal disease have decreased bioactivity of insulin-like growth factor (IGF-1), whose activity significantly decreases urinary glutamate levels^26^.

The third metabolite identified as a possible LN biomarker is glycolic acid, a secondary bile acid. Several studies have reported that high bile acid plasma levels are associated with chronic kidney disease (CKD) progression. However, decreased bile acid levels have been reported in urine samples from patients with CKD, similar to our study^20^. The bile acid concentration in blood and urine depends on glomerular filtration performed through apical sodium-dependent bile acid transporters, multidrug resistance associated protein 2 (MRP2), and the organic solute transporters alpha and beta. The pathological changes observed in patients with CKD—such as mesangial and endothelial cell proliferation, glomerular sclerosis, renal interstitial fibrosis, and intrarenal vascular sclerosis—reduce glomerular filtration; thus, bile acid filtration tends to decrease in patients with renal diseases^29,30^.

In our study, MSEA was conducted using the KEGG pathway database. This approach also allowed for the identification of the metabolic pathways associated with kidney damage progression in patients with SLE, which include primary bile acid biosynthesis, BCAA synthesis and catabolism, and tryptophan metabolism. Primary bile acid biosynthesis has been associated with the lipid profile alterations typically observed in patients with SLE^31,32^. In this case, high cholesterol and glycosphingolipid levels in the T-cell membrane change the composition of signaling platforms, thus favoring proinflammatory signaling^19,33^. Moreover, as discussed above, lipid metabolism alterations are associated with lipid nephrotoxicity and its role in LN pathophysiology^34^. Certain bile acids, such as deoxycholic acid, glycolic acid, ursodeoxycholic acid, and arachidonic acid, are significantly correlated with patients’ systemic lupus erythematosus disease activity index (SLEDAI) score and have shown adequate power to predict disease activity^31^.

In addition to their role in lipid metabolism, bile acids are signaling molecules that act through the activation of bile acid receptors. A study reported decreased levels of farnesoid X receptors (FXRs) in patients with SLE and murine MRL/lpr models of lupus and hepatic failure. In this study, the use of chenodeoxycholic acid, an agonist of FXR, suppressed the expression of inflammatory cytokines such as TNF-α, interferon-gamma (IFN-γ), and interleukin 6 (IL-6) in mice^34^. Another study revealed the modulatory effect of bile acids on intestinal immunity and showed that metabolites derived from lithocholic acid (LCA), 3-oxoLCA, and isoallolLCA can inhibit Th17 differentiation by directly binding with the key transcription factor retinoid-related orphan receptor γt (RORγt). Moreover, these metabolites improve FOXP3 gene expression by producing mitochondrial ROS (mitoROS), which results in regulatory T-cell expansion^35,36^.

The role of BCAAs in immunity is mediated through the phosphoinositide 3-kinase-protein kinase B-mammalian target of rapamycin (PI3K/AKT/mTOR) signaling pathway^37^. Mammalian target of rapamycin (mTOR) activity is regulated by amino acid availability, energy levels, and growth factors. In mammalian cells, mTOR forms two different complexes: mTORC1 and mTORC2. mTORC1 detects various stress signals, including the accumulation of amino acids such as leucine, isoleucine, kynurenine, and glutamine. mTORC activity increases in Th17 cells and T cells that produce IL-4, leading to the proinflammatory profile observed in patients with SLE. mTOR is required for cell differentiation toward the Th17 subtype through the induction of hypoxia-inducible factor 1α (HIF1α), which enhances glycolysis in inflammatory cells during the pseudohypoxia that typically occurs in patients with SLE^38,39^.

BCAA catabolism initially occurs through transamination by aminotransferases (BCAT) or decarboxylation by the branched-chain α-ketoacid dehydrogenase complex (BCKDC). Following these reactions, BCAA metabolites turn into acetyl-CoA and succinyl-CoA and participate in the tricarboxylic cycle (TCA cycle). In CD4^+^ T cells, BCAT negatively regulates mTOR and glycolysis. Activated T cells from mice with branched-chain amino acid aminotransferase (BCATc) deficiency show an increase in mTORC1 activation compared with the T cells from control mice^39^. In addition, another study reported that oral administration of ERG240, an analog of leucine, selectively inhibited BCAT1 activity, thus reducing the severity of collagen-induced arthritis and extracapillary proliferative glomerulonephritis in mice^40^.

To date, most of the amino acids analyzed in peripheral blood samples of patients with SLE, including gluconeogenic and ketogenic amino acids, have shown decreased levels. Ammonia is the catabolic product of amino groups; it can be converted to urea through the urea cycle and is subsequently excreted in the urine. A metabolomics study measured the metabolites associated with the urea cycle and revealed that both arginine, the immediate precursor of urea, and urea itself were increased in patients with SLE, thus suggesting increased activity in the urea cycle^41,42^. The kidneys play a significant role in amino acid homeostasis. In studies conducted with kidneys from LMR/lpr mice with SLE, BCAA concentrations were altered, which may suggest decreased protein synthesis, increased protein degradation, or both. The kidney is a dynamic organ with various enzymatic machinery components for amino acid catabolism and/or oxidization, particularly in the ascending limb of the loop of Henle, which provides the necessary energy for active ion transportation. The observed alterations in amino acid levels may be linked to the regulation of gene transcription, cell cycle progression, and immune and inflammatory responses. In the context of LN, these metabolic shifts could be a response to the underlying pathology, potentially reflecting an attempt by the body to regulate various cellular processes in the face of immune system dysregulation and inflammation^43,44^.

The LN samples analyzed in our study had higher amino acid levels than did the SLE samples. Patients with LN had higher concentrations of BCAAs and their metabolites, such as valine (SLE vs. LN: fold change = 5.81), hydroxyisoleucine (SLE vs. LN: fold change = 37.13), and aminoadipic acid (SLE vs. LN: fold change = 335). Therefore, the alteration of these metabolites and their metabolic pathways can be associated with renal disease progression in patients with SLE.

Finally, multiple mechanisms underlying the role of tryptophan in lupus progression have been proposed. Several studies have described biased metabolism of tryptophan toward the kynurenine pathway in patients with SLE, which is reflected by low tryptophan concentrations and high kynurenine levels in the serum of these patients^45^. Prior studies have shown that exogenous kynurenine enhances Th1 polarization of CD4^+^ T cells and reduces Treg cell polarization of cytotoxic T cells, thus suggesting that kynurenine promotes proinflammatory T-cell phenotypes. Moreover, kynurenine induces the activation of mTOR in human T cells^46^, contributing to the high level of mTOR activation typically observed in the CD4^+^ T cells of patients with SLE. In CD4^+^ T cells, active gene hypomethylation in the mTOR pathway increases the expression and activation of proinflammatory cytokines such as IFNγ and IL-17^47^, which are key for SLE pathophysiology. Studies conducted with mice have proven the efficacy of mTOR inhibition by rapamycin for treating LN in children. mTOR inhibition by rapamycin reduces STAT3 activation in effector T cells, as well as the migration of IL-17-producing T cells in inflamed kidneys, thus eliminating chronic inflammatory processes^48^.

Moreover, several tryptophan-derived metabolites, including indole-3-aldehyde, indol-3-acetic acid, 3-metilindole, tryptamine, and indoxyl sulfate, are ligands for aryl hydrocarbon receptors (AhRs). AhR signaling modulates many essential cell processes, such as cell cycle progression, apoptosis, and cell proliferation, by regulating P53, FasR, Bcl-2, and kinases of the cell cycle. AhR activation increases the regulation of genes encoding cytokines, such as IL-10, which regulate immune tolerance^35,45^. In patients with SLE, exposure to indoxyl sulfate, a metabolite of tryptophan degradation, increases AhR activity in the periglomerular region and in the proximal and distal renal tubules, causing renal fibrosis characterized by podocyte injury, progressive glomerular damage, and a proinflammatory phenotype associated with LN^39^.

The metabolic pathway of tryptophan and its particular relationship with LN have also been studied. In a metabolomics study conducted with urine samples to identify possible metabolites associated with membranous LNs, the picolinic acid–tryptophan ratio had considerable potential for LN diagnosis and classification. Therefore, the metabolites of this pathway are currently being considered potential alternative biomarkers for the noninvasive diagnosis of LN^49^. Our study showed a significant difference in the hydroxyanthranilic acid levels between samples from patients with LES and patients with LN (fold change = 0.66). Hydroxyanthranilic acid is produced by the metabolism of tryptophan through the kynurenine pathway, revealing the role of this pathway in LN occurrence. Several studies have reported high levels of hydroxyanthranilic acid in the plasma of patients with CKD. However, its renal excretion is limited under disease conditions; thus, its urinary concentration tends to decrease in patients with glomerular disease, as this study showed^50^.

Despite these findings, few studies have identified metabolic changes between SLE patients and LN patients. A study published by Guleria et al., which used 1H magnetic resonance spectroscopy, reported that patients with LN had higher serum lipid (LDL/very LDL) and creatinine levels and lower acetate levels than patients with SLE^51^. In general, studies aimed at determining the metabolomics profile of patients with SLE and LN based on urine samples are scarce. Although urine is an excellent option for identifying biomarkers of LN because it emerges directly from the affected renal tissue and is the most accurate biological fluid reflecting kidney dynamics^4,52^, a few studies have been conducted so far using this biofluid. In two of these studies, performed by Guleria et al.^51^, and Ganguly et al.^53^, a significant reduction in serum or urine levels of citrate was observed, when compared to healthy controls. In general, the behaviour of citrate in urine and serum is similar since it passes freely through the glomerulus, 60% of it being reabsorbed in the proximal tubule. Citrate is a tricarboxylic acid synthetized in the mitochondria that plays a key role in the TCA—therefore, it is reasonable to expect that when immune cells are activated and their energetic metabolism shifts, its levels in serum decrease, as the oxidative phosphorylation diminishes^53^. Acetate also showed significant changes in these two studies. Acetate is a product of the oxidation of fatty acids, and it was reported to be elevated in the serum samples of LN patients, compared to HC, which support the theory of disturbed lipid metabolism in LN patients^43,51^. Ganguly et al.^53^ observed higher acetate levels in LN, which exhibited a decreasing trend after treatment, possibly indicating tubular repair. Fatty acid oxidation primarily occurs in the mitochondria and peroxisomes of nephron tubules, especially the proximal tubules. Furthermore, toxins damaging the proximal tubules may lead to increased acetate excretion in urine, potentially explaining the differential behavior of acetate in serum and urine. Thanks to these study it has been demonstrated that factors such as the renal processes of filtration, reabsorption, and secretion of biomarkers, as well as the activity of active transporters in the kidneys can impact the levels of specific biomarkers in urine samples compared to serum or plasma. The behavior of these transporters may reflect changes in metabolic pathways or disruptions in kidney function, thereby establishing urine biomarkers as valuable indicators in metabolomics, especially for kidney related diseases.

In understanding results from metabolomics studies of diseases like SLE and LN, it's crucial to remember that factors beyond the disease itself can significantly impact the levels of metabolites. Medications commonly used for these conditions, such as glucocorticoids and immunosuppressants, are a major group of these influencing factors. Glucocorticoids, which are almost always used for disease control, can unfortunately bring unintended consequences, and it has been demonstrated how they affect metabolism, including imbalances in glucose, lipids, and proteins^41^.

In an experiment performed by Malkawi et al^54^, where rats were treated with Dexamethasone at a dose of 2.5 mg/kg twice a week for 14 weeks, it was demonstrated that the serum metabolome was characterized by a decrease in phenylalanine, lysine, and arginine, while levels of tyrosine, hydroxyproline, and acylcarnitines were increased. These changes suggest that Dexamethaose may affect processes like the production of glucose from non-carbohydrates, the breakdown of proteins, and the breakdown of fat tissue. A similar study was performed in healthy volunteers, where a single 4-mg dose of dexamethasone caused major changes in over 150 plasma metabolites, characterized by an increase of blood sugar, lactate, mannose, and some amino acids, and a decrease in of cholesterol and fatty acids^55^. Interestingly, in another study performed with patients with Cushing’s syndrome or adrenocortical adenomas with or without hypercortisolism that were compared with hormonally normal controls, those with high cortisol had lower levels of certain fats and amino acids, but higher levels of polyamines. This suggests that some of the metabolic changes in SLE might be caused by the body's own natural steroids, while others might not be as affected by these hormones^56^.

Additionally, a study found no clear link between taking glucocorticoids and changes in the blood related to oxidative stress, production of glutathione and specific inflammatory pathways. This includes substances like MDA, glutathione itself, leukotriene B4, and gamma-glutamyltransferase 1 (GGT1)^41,42^.

Unlike other immunosuppressant medications used for SLE, hydroxychloroquine (HCQ) seems to have a beneficial effect on cholesterol and blood sugar^41^. Studies have shown that HCQ induces the decrease of low density lipoprotein, triglycerides, and very low-density lipoprotein^57,58^. On the other hand, medications like cyclosporine and tacrolimus have been reported to worsen cholesterol and blood sugar levels^59^. These negative effects are dose-dependent, meaning the higher the dose, the worse the impact, and can eventually lead to hyperglycemia and hyperlipidemia^60^. Thankfully, azathioprine and cyclophosphamide, other SLE medications, seem to have no influence in the metabolome of SLE patients^41^.

Since medications can affect the results of metabolic studies in SLE and LN patients, it's crucial to consider their influence before drawing conclusions. To get a clearer picture of how lupus itself affects metabolism, future studies should ideally involve patients who haven't received any medications yet (drug-naive, new-onset SLE). Additionally, studying metabolism in mice with lupus might provide valuable insights in this area. In our study, due to the limitations in the data regarding medications used by SLE and LN patients, including the specific medications, dosages, and treatment durations, it is not possible to definitively determine or precisely estimate the impact of these treatments on the observed metabolic changes. However, this important consideration will be factored into the design of future research to ensure a more accurate understanding of the metabolic effects of SLE and LN independent of medication influence. Because of the complex pathophysiology of SLE and the difficulty in reaching an adequate diagnosis, metabolomics may be a good alternative approach for identifying new noninvasive biomarkers. However, multiple confounding factors may arise in metabolomics studies, which results in study limitations. We believe that factors such as participants’ concomitant medications, eating habits, alcohol use, smoking, and consumption of other substances cause the heterogeneity of the data collected during analysis. Therefore, in our upcoming studies, we aimed to control for these confounding factors and expand the sample size, including newly diagnosed patients, to compare subgroups based on clinical data, including the SLEDAI score, sex, and treatment. Moreover, validating the metabolites with the highest predictive power through targeted metabolomics utilizing standardized references, will be highly valuable for confirming our findings and proposing these metabolites as potential biomarkers for LN diagnosis.

## Implications for clinical practice

This research successfully identified promising markers for diagnosing lupus nephritis. These markers are particularly valuable for personalized medicine because ideally, they could be detected through simple urine tests. Urine is especially attractive for research due to its ease of collection, making it a treasure trove of potential information for future studies. Analyzing the unique chemical fingerprints found in urine is a novel and exciting approach compared to traditional methods. As our understanding of these metabolites and how they connect to health and medications expands, they could become powerful tools for developing personalized treatments for lupus nephritis patients. Ultimately, validating these potential markers, and identifying the best ones, could revolutionize how we diagnose and treat lupus related kidney diseases. By using painless urine tests and enabling personalized treatment plans, this research paves the way for improved patient comfort and overall quality of care.

### Supplementary Information

Supplementary Information 1.

Supplementary Information 2.

## Data Availability

Our raw data will be deposited in the Metabolights database (https://www.ebi.ac.uk/metabolights/) and will be made available once it has been uploaded.
