# Supplementary material for: Urinary metabolomic profiling of a cohort of Colombian patients with systemic lupus erythematosus
Source: Sci Rep. 2024 Apr 25;14:9555. doi: 10.1038/s41598-024-60217-0 (PMC11045835; doi:10.1038/s41598-024-60217-0)
Supplement: Supplementary file 2 — Supplementary Information 2. [file 41598_2024_60217_MOESM2_ESM.docx]

Supplementary material:


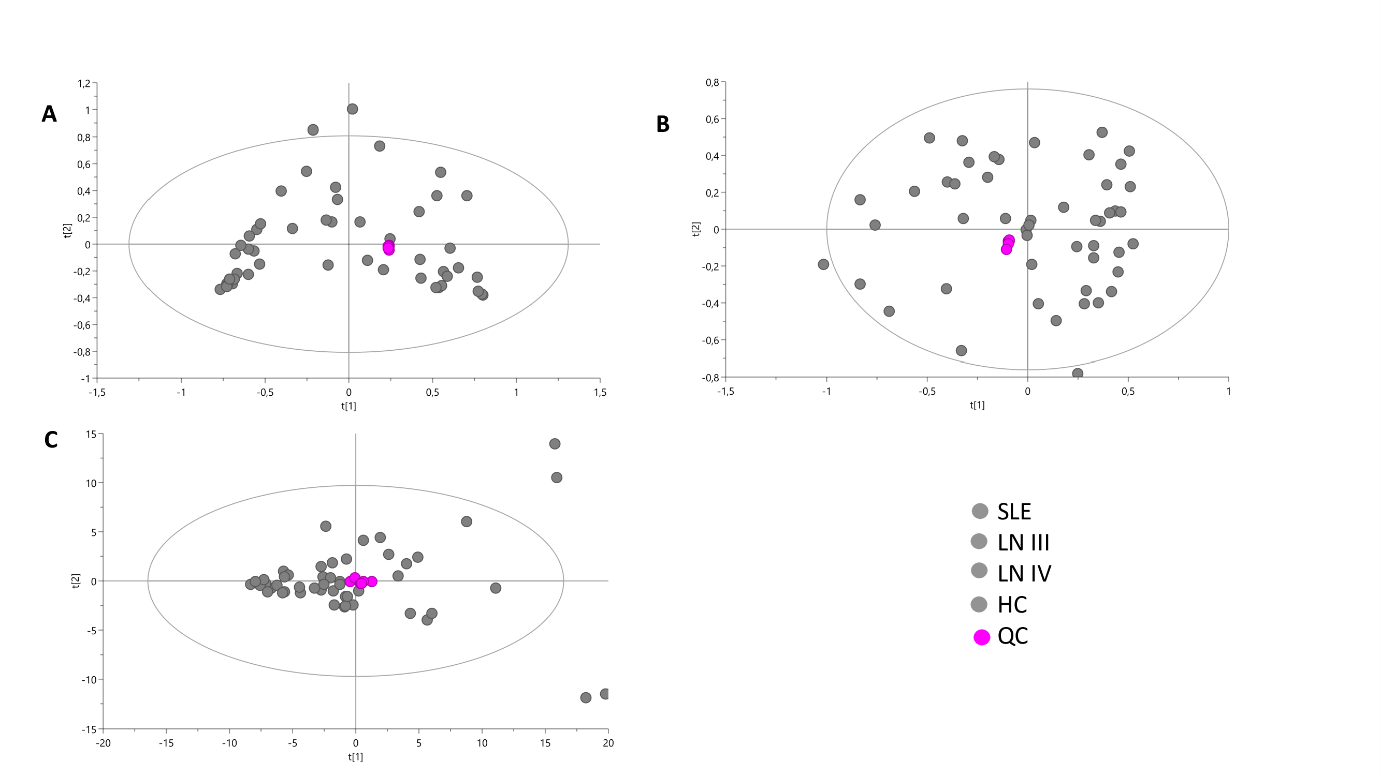


Supplementary Figure 1: PCA scatter plots for the dataset. Assessment of data quality through the clustering of quality control samples (magenta points: quality control; gray points: group of healthy controls, SLE, LN class III, and LN class IV). A. Untargeted metabolomics via RP-LC/MS-QTOF (+): R^2^ = 0.682, Q^2^ = 0.341. B. Untargeted metabolomics via RP-LC/MS-QTOF (-): R^2^ = 0.687, Q^2^ = 0.309. C. Untargeted metabolomics via GC-MS: R^2^ = 0.721, Q^2^ = 0.05.

| **Compound** | **Formula** | **Mass** | **RT (min)** | **Mass Error (ppm)** | **Adduct** | **^a^CV for QC (%)** | **Analytical platform** | **DET** | **^b^ID level** | **^c^Fold Change** | **^d^VIP** | **^e^ρ value*** |
| --- | --- | --- | --- | --- | --- | --- | --- | --- | --- | --- | --- | --- |
| **Amino acids and analogues** | | | | | | | | | | | | |
| Creatinine | C_4_H_7_N_3_O | 113.0589 | 0.32 | 5 | M+H | 7.11 | LC-QTOF-MS | ESI+ | 3 | 0.63 | 1.49 | 3.85E-03* |
| Proline betaine | C_7_H_13_NO_2_ | 143.0946 | 0.35 | 3 | M+H | 6.53 | LC-QTOF-MS | ESI+ | 3 | 2.29 | 2.21 | 1.48E-02* |
| Hydroxy isoleucine | C_6_H_13_NO_3_ | 147.0895 | 0.36 | 6 | M+H | 10.24 | LC-QTOF-MS | ESI+ | 3 | 37.13 | 1.94 | 2.95E-04* |
| Pipecolic acid | C_6_H_11_NO_2_ | 129.1573 | 11.27 | - | - | 6.11 | GC-QTOF-MS | - | 2 | 2.21 | - | 1.38E-02 |
| Proline | C_5_H_9_NO_2_ | 115.1306 | 10.35 | - | - | 13.10 | GC-QTOF-MS | - | 2 | 3.64 | - | 2.004E-02 |
| Acetyl-aspartic acid | C_6_H_9_NO_5_ | 175.1396 | 14.86 | - | - | 11.24 | GC-QTOF-MS | - | 2 | 0.68 | - | 3.75 E-02 |
| Aminolevulinic acid | C_5_H_9_NO_3_ | 131.1301 | 9.92 | - | - | 9.31 | GC-QTOF-MS | - | 2 | 0.58 | 1.38 | - |
| Acetyl-glutamic acid | C_7_H_11_NO_5_ | 189.1662 | 16.24 | - | - | 15.30 | GC-QTOF-MS | - | 2 | 0.58 | 1.41 | - |
| methyl-glutamic Acid | C_6_H_11_NO_4_ | 161.1561 | 13.13 | - | - | 28.24 | GC-QTOF-MS | - | 2 | 4292.06 | 1.99 | 6.49E-06* |
| Methyl-proline | C_6_H_11_NO_2_ | 129.1573 | 8.46 | - | - | 27.82 | GC-QTOF-MS | - | 2 | 4.40 | - | 4.34E-03* |
| Amino-phosphonopropionic acid | C_3_H_8_NO_5_P | 169.0140 | 0.48 | 7 | M+H | 9.91 | LC-QTOF-MS | ESI+ | 3 | 0.54 | 1.93 | 8.60E-03* |
| Valine | C_5_H_11_NO_2_ | 117.0790 | 0.49 | 6 | M+H | 11.63 | LC-QTOF-MS | ESI+ | 3 | 5.81 | 1.98 | 3.82E-03* |
| Aminoadipic acid | C_6_H_11_NO_4_ | 161.0688 | 0.49 | 4 | M+H | 10.66 | LC-QTOF-MS | ESI+ | 3 | 335 | 1.69 | 2.65E-06* |
| Leucyl proline | C_11_H_20_N_2_O_3_ | 228.1474 | 0.51 | 3 | M+H | 3.30 | LC-QTOF-MS | ESI+ | 3 | 0.64 | 2.34 | 2.41E-04* |
| Glutamine | C_5_H_10_N_2_O_3_ | 146.0691 | 3.69 | 2 | M-H | 1.48 | LC-QTOF-MS | ESI- | 2 | 0.76 | 3.30 | 2.13E-02* |
| Pyroglutamic acid | C_5_H_7_NO_3_ | 129.0426 | 3.72 | 4 | M+H | 5.19 | LC-QTOF-MS | ESI+ | 2 | 0.60 | 2.99 | 2.64E-03* |
| Phenylacetylglutamine | C_13_H_16_N_2_O_4_ | 264.1110 | 3.72 | 4 | M+H | 7.79 | LC-QTOF-MS | ESI+ | 2 | 0.57 | 1.89 | 4.09E-03* |
| **Benzenoids** | | | | | | | | | | | | |
| Phenol | C_6_H_6_O | 94.0419 | 3.39 | 8 | M+H | 2.93 | LC-QTOF-MS | ESI+ | 3 | 0.55 | 2.84 | 1.06E-03* |
| Benzoic acid | C_7_H_6_O_2_ | 122.0368 | 3.39 | 5 | M+H | 4.92 | LC-QTOF-MS | ESI+ | 3 | 0.56 | 2.77 | 2.14E-06* |
| Methyl benzaldehyde | C_8_H_8_O | 120.0575 | 7.21 | 5 | M+H | 2.28 | LC-QTOF-MS | ESI+ | 3 | 25.73 | 1.33 | 1.87E-04* |
| Hydroxybenzyl amine | C_7_H_9_NO | 123.0684 | 5.99 | 4 | M+H | 8.91 | LC-QTOF-MS | ESI+ | 3 | 0.003 | 1.09 | 2.85E-06* |
| Hydroxy anthranilic acid | C_18_H_20_N_2_O_8_ | 153,0426 | 0.95 | 2 | M+H | 5.03 | LC-QTOF-MS | ESI+ | 4 | 0.66 | 1.23 | 1.83E-03* |
| **Bile acids, alcohols, and derivatives** | | | | | | | | | | | | |
| Glycocholic acid | C_26_H_43_NO_6_ | 465.3090 | 9.25 | 1 | M-H | 3.89 | LC-QTOF-MS | ESI- | 3 | 0.13 | 1.03 | 4.93E-05* |
| **Carbohydrates and carbohydrate conjugates** | | | | | | | | | | | | |
| Mycophenolic acid O-acyl-glucuronide | C_23_H_28_O_12_ | 496.1581 | 7.24 | 2 | M-H | 0.68 | LC-QTOF-MS | ESI- | 3 | 1446 | 9.75 | 2.69E-04* |
| Ribose | C_5_H_10_O_5_ | 150.1301 | 15.50 | - | - | 5.43 | GC-QTOF-MS | - | 2 | 0.59 | - | 2.66E-02 |
| Deoxy-ribose | C_5_H_10_O_4_ | 134.1307 | 13.58 | - | - | 13.95 | GC-QTOF-MS | - | 2 | 0.37 | 1.67 | 2.66E-02 |
| Gluconic acid | C_6_H_12_O_7_ | 196.0583 | 2.81 | 4 | M-H | 7.68 | LC-QTOF-MS | ESI- | 3 | 0.34 | 1.17 | 5.13E-03* |
| **Fatty Acyls** | | | | | | | | | | | | |
| hydroxynonanoyl carnitine | C_16_H_31_NO_5_ | 317.2202 | 7.32 | 4 | M+H | 3.96 | LC-QTOF-MS | ESI+ | 2 | 0.46 | 1.66 | 1.21E-02* |
| Nonanoylcarnitine | C_16_H_31_NO_4_ | 301.2253 | 7.87 | 5 | M+H | 3.96 | LC-QTOF-MS | ESI+ | 2 | 0.36 | 3.04 | 1.42E-03* |
| decatrienoylcarnitine | C_17_H_27_NO_4_ | 309.1940 | 7.54 | 5 | M+H | 8.54 | LC-QTOF-MS | ESI+ | 2 | 0.48 | 1.14 | 2.77E-02* |
| Erucamide | C_22_H_43_NO | 337.3345 | 11.33 | 6 | M+H | 10.04 | LC-QTOF-MS | ESI+ | 2 | 0.63 | 1.36 | 4.92E-03* |
| ketopalmitoyl-CoA | C_37_H_64_N_7_O_18_P_3_S | 1019.3241 | 11.76 | 4 | M+H | 10.90 | LC-QTOF-MS | ESI+ | 4 | 0.55 | 1.30 | 2.94E-03* |
| Feruloylglucose trihydroxy-methylbutylglycoside | C_21_H_30_O_12_ | 474.1737 | 7.25 | 9 | M+H | 3.37 | LC-QTOF-MS | ESI+ | 4 | 1005 | 3.01 | 1.43E-06* |
| oxo-pentenoic acid | C_5_H_6_O_3_ | 114.0317 | 7.24 | 5 | M-H | 1.58 | LC-QTOF-MS | ESI- | 4 | 15.02 | 1.96 | 4.12E-06* |
| Itaconic acid | C_5_H_6_O_4_ | 130.0266 | 0.50 | 5 | M-H | 3.47 | LC-QTOF-MS | ESI- | 2 | 0.47 | 1.07 | 4.48E-02* |
| **Glycerophospholipids** | | | | | | | | | | | | |
| LPE 18:4 | C_23_H_40_NO_7_P | 473.2542 | 3.67 | 0 | M+H | 5.89 | LC-QTOF-MS | ESI+ | 3 | 297 | 1.70 | 2.66E-06* |
| **Hydroxy acids and derivatives** | | | | | | | | | | | | |
| (Methylthio) hexylmalic acid | C_11_H_20_O_5_S | 264.1031 | 4.10 | 2 | M+H | 7.78 | LC-QTOF-MS | ESI+ | 3 | 29.94 | 1.46 | 9.78E-06* |
| **Indoles and derivatives** | | | | | | | | | | | | |
| (Indol-ylacetyl-myo-inositol)-galactoside | C_22_H_29_NO_12_ | 499.1690 | 4.32 | 4 | M+H | 7.24 | LC-QTOF-MS | ESI+ | 3 | 0.09 | 1.69 | 2.92E-05* |
| **Organic acids and derivatives** | | | | | | | | | | | | |
| Ethyl  (methyldithio)propionate | C_6_H_12_O_2_S_2_ | 180.0278 | 4.10 | 2 | M+H | 6.57 | LC-QTOF-MS | ESI+ | 3 | 611 | 1.60 | 1.43E-06* |
| Thiobispropanoic acid | C_6_H_10_O_4_S | 178.0300 | 4.10 | 4 | M+H | 5.41 | LC-QTOF-MS | ESI+ | 3 | 1878 | 2.25 | 1.43E-06* |
| Phenol sulphate | C_6_H_6_O_4_S | 173.9987 | 1.72 | 3 | M-H | 3.26 | LC-QTOF-MS | ESI- | 3 | 0.16 | 1.40 | 1.58E-03* |
| **Organic disulfides** | | | | | | | | | | | | |
| Methyl methyl-butenyl disulfide | C_6_H_12_S_2_ | 148.0380 | 1.92 | 7 | M+H | 2.34 | LC-QTOF-MS | ESI+ | 3 | 0.45 | 1.43 | 5.00E-05* |
| **Phenylpropanoids** | | | | | | | | | | | | |
| Ferulic acid | C_10_H_10_O_4_ | 194.0579 | 7.25 | 3 | M+H | 1.65 | LC-QTOF-MS | ESI+ | 3 | 312 | 3.46 | 1.01E-03* |
| Benzylsuccinate | C_11_H_12_O_4_ | 208.0736 | 3.93 | 5 | M+H | 16.65 | LC-QTOF-MS | ESI+ | 3 | 0.01 | 1.03 | 7.32E-06* |
| **Prostaglandins** | | | | | | | | | | | | |
| phenyl-trinor-PGF2alpha isopropyl ester | C_26_H_38_O_5_ | 430.2719 | 9.26 | 5 | M+H | 10.03 | LC-QTOF-MS | ESI+ | 3 | 0.08 | 1.01 | 1.17E-05* |
| **Purines and purine derivatives** | | | | | | | | | | | | |
| Methyladenine | C_6_H_7_N_5_ | 149.0701 | 0.55 | 1 | M+H | 3.63 | LC-QTOF-MS | ESI+ | 2 | 0.53 | 1.05 | 4.32E-04* |
| **Pteridines and derivatives** | | | | | | | | | | | | |
| Pteridine | C_6_H_4_N_4_ | 132.0436 | 5.32 | 8 | M-H | 4.30 | LC-QTOF-MS | ESI- | 3 | 0.24 | 1.22 | 2.86E-04* |
| **Organic acids** | | | | | | | | | | | | |
| Ketoglutaric acid | C_5_H_6_O_5_ | 146.0983 | 13.93 | - | - | 3.73 | GC-QTOF-MS | - | 2 | 0.63 | - | 3.28E-02 |
| **-Carboxylic acids** | | | | | | | | | | | | |
| Maleic acid | C_4_H_4_O_4_ | 116.0723 | 10.27 | - | - | 13.14 | GC-QTOF-MS | - | 2 | 0.49 | 1.45 | - |
| **Glycerolipids** | | | | | | | | | | | | |
| Monopalmitin | C_19_H_38_O_4_ | 330.5033 | 23.44 | - | - | 6.33 | GC-QTOF-MS | - | 2 | 4.19 | 2.50 | 6.49E-06* |
| Palmitoyl glycerol | C_19_H_38_O_4_ | 330.5033 | 23.18 | - | - | 12.08 | GC-QTOF-MS | - | 2 | 4.30 | 2.40 | 9.12E-06* |
| **Nucleosides** | | | | | | | | | | | | |
| Uridine | C_9_H_12_N_2_O_6_ | 244.2018 | 22.10 | - | - | 5.16 | GC-QTOF-MS | - | 2 | 0.71 | 1.10 | - |

**Supplementary table 1.** Differential metabolites found between SLE and LN III/IV. RT: retention time; aCV, coefficient of variation in the metabolites in the QC samples; bIdentification level: Level 1 Structure confirmed, Level 2 Structure probable, Level 3 Unequivocal molecular formula or MSMS data (s), Level 4 Exact mass; cFold Change, change in the abundance of the specified comparison calculated as (case/control); dVIP, variable importance in projection. ep value* correspond to the p values calculated by the Benjamini-Hochberg false discovery rate post hoc correction (FDR < 0.05); GM: global metabolomics, LC: liquid chromatography, GC: gas chromatography, QTOF-MS: quadrupole time-of-flight mass spectrometer.
